# The Effects of Home High-Flow Nasal Cannula Oxygen Therapy on Clinical Outcomes in Patients with Severe COPD and Frequent Exacerbations

**DOI:** 10.3390/jcm14030868

**Published:** 2025-01-28

**Authors:** Christiaan Theunisse, Netty T. C. de Graaf, Annemiek W. E. Braam, Greet C. Vonk, Sara J. Baart, Huibert H. Ponssen, David Cheung

**Affiliations:** 1 Department of Pulmonology, Albert Schweitzer Hospital, NL-3318 AT Dordrecht, The Netherlands; c.theunisse@asz.nl (C.T.); n.degraaf@asz.nl (N.T.C.d.G.); 2 Department of Intensive Care, Albert Schweitzer Hospital, NL-3318 AT Dordrecht, The Netherlands; h.ponssen@erasmuc.nl; 3 Department of Pulmonology, Beatrix Hospital, 3311 JX Gorinchem, The Netherlands; annemiekbraam@icloud.com (A.W.E.B.); g.vonk@rivas.nl (G.C.V.); 4 Department of Biostatics, Erasmus University Medical Center, 3015 GD Rotterdam, The Netherlands; s.baart@erasmusmc.nl

**Keywords:** COPD, high flow nasal cannula, domiciliary, hospitalization, COPD exacerbations

## Abstract

**Background:** Chronic Obstructive Pulmonary Disease (COPD) is a disease with high morbidity and mortality globally. Exacerbations of COPD are major contributors to disease progression and a decline in health-related quality of life (HRQoL). High-flow nasal cannula (HFNC) oxygen therapy is an innovative therapy that provides humidified and heated blended air and oxygen through a nasal cannula. There is some preliminary evidence supporting the effectiveness of HFNC in managing COPD exacerbations, but there are limited data on its effectiveness when used at home for patients with stable, severe COPD. The aim of the present study is to test the hypothesis that home HFNC can decrease the COPD exacerbations rate and hospital admissions and improve HRQoL measures in severe COPD patients with frequent COPD exacerbations. **Methods:** In a prospective proof-of-concept interventional multicenter study, 40 GOLD stage III and IV COPD patients with a high disease burden (≥2 exacerbations treated with antibiotics and/or corticosteroids) and ≥1 hospital admission in the last year were included. Patients were given instructions on the usage of HFNC by a ventilation practitioner during a group session. The flow rate was 25–30 L/min and FiO_2_ was 21–35%. Outcome measures included the COPD exacerbations rate, hospital admissions, in-hospital days, Medical Research Council dyspnea (MRC) score, Clinical COPD Questionnaire (CCQ) score, Hospital Anxiety Depression Scale (HADS) scores and capillary pCO_2_. Repeated analysis of variance (ANOVA) was used to analyze the data. Significant effects identified in the ANOVA were further examined using Student’s *t*-tests. **Results:** After 1 year, 27 patients could be evaluated. The COPD exacerbations rate decreased by 1.40 (mean difference ± SD: 1.40 ± 2.09; *p* = 0.002), hospital admissions decreased by 0.96 admissions per year (0.96 ± 1.37; *p* = 0.001), and in-hospital days decreased by 7.22 days (7.22 ± 9.26; *p* = 0.001). Capillary pCO_2_ decreased by 0.02 kPa (0.02 ± 0.52; *p* = 0.85). The CCQ score decreased by 0.06 (0.06 ± 0.96; *p* = 0.76). The MRC dyspnea score decreased by 0.04 (0.04 ± 0.80; *p* = 0.81). The HADS anxiety score decreased by 0.63 (0.63 ± 3.12; *p* = 0.31). And finally, the HADS depression score decreased by 0.32 (0.32 ± 3.48; *p* = 0.64). There was a significant difference between the normocapnic (capillary pCO_2_ < 6.0 kPa) group and the hypercapnic group in terms of change in the CCQ score (−0.24 ± 0.55 and 0.49 ± 1.32 decrease, respectively, *p* = 0.05) and the HADS depression score (−0.76 ± 1.86 and 2.20 ± 4.75 decrease, respectively, *p* = 0.03) after 1 year of HFNC treatment. **Conclusions:** One-year-long HFNC therapy significantly decreased the COPD exacerbations rate, hospital admissions, and in-hospital days in severe COPD patients with a high disease burden and frequent COPD exacerbations irrespective of them having hypercapnia and with the HRQoL measures only improving in the hypercapnic group. This may imply that severe COPD patients with a high disease burden and frequent COPD exacerbations, irrespective being hypercapnic, are candidates for treatment with home HFNC oxygen therapy.

## 1. Introduction

Chronic Obstructive Pulmonary Disease (COPD) is a progressive lung disease characterized by persistent airflow obstruction and frequent exacerbations [1]. The management of COPD typically involves pharmacological treatments, such as bronchodilators and corticosteroids, pulmonary rehabilitation and, in some cases, long-term oxygen therapy for those with severe disease. Exacerbations play a significant role in the increased morbidity, mortality, and diminished health status of COPD patients, placing a considerable burden on healthcare systems [2]. Around 15% of COPD patients experience exacerbations that necessitate hospitalization each year [3,4]. As a result, minimizing the frequency of exacerbations and hospital admissions is a key therapeutic goal. The body of evidence supporting the use of home non-invasive ventilation (NIV) for severe COPD patients with persistent hypercapnic respiratory failure is increasing [5,6], and respiratory societies have issued guidelines recommending its use in such cases [7,8]. Long-term use of home NIV has been shown to offer several benefits, including improved survival, a reduced frequency of COPD exacerbations, fewer hospitalizations, and an enhanced quality of life [5]. However, NIV can be challenging for many patients to tolerate, with some experiencing discomfort or difficulty adjusting to the treatment [9]. High-flow nasal cannula (HFNC) oxygen therapy is an innovative and straightforward gas delivery system composed of an air–oxygen blender, active heated humidifier, single heated circuit and nasal cannula and can deliver high-rate humidified oxygen (up to 60 L/min) through a nasal cannula [10]. Several studies have demonstrated that the comfort and tolerance associated with HFNC is significantly higher than that of NIV [11]. Initially, HFNC therapy was established in acute and critical care settings for treating mild-to-moderate acute hypoxic failure [12] and ventilator weaning [13]. HFNC therapy has been shown to be as effective as NIV in preventing post-extubation respiratory failure or re-intubation in patients with hypoxemic respiratory failure [14]. More recently, increasing evidence suggests that HFNC therapy is also beneficial in treating acute exacerbations of COPD [15]. Short-term use of an HFNC has been demonstrated to improve breathing patterns, decrease inspiratory effort, and reduce hypercapnia in patients with stable hypercapnic COPD, due to its physiological effects [16,17]. However, there is limited knowledge regarding the long-term effects of HFNC therapy in patients with stable COPD. Recently, a pivotal study on the long-term effect of HFNC therapy by Nagata et al. showed that HFNC therapy significantly reduced the number of exacerbations and prolonged the time to the first exacerbation in severe COPD patients with hypercapnia [18]. However, it remains unclear whether long-term HFNC can also be effective for patients with severe COPD without hypercapnia. We hypothesize that in patients with severe COPD (GOLD stages III and IV) with a high disease burden and frequent exacerbations, regardless of the presence of hypercapnia, long-term HFNC will reduce the rate of exacerbations and hospitalizations and improve health-related quality of life (HRQoL). The aim of this study was to investigate, as a proof of concept, whether HFNC therapy can enhance quality of life of COPD patients and reduce the frequency of COPD exacerbations and hospitalizations in patients with severe COPD with frequent exacerbations, irrespective of hypercapnia. 

## 2. Material and Methods

### 2.1. Ethics

The study protocol was approved by the local ethics committee of Albert Schweitzer Hospital Dordrecht, The Netherlands (study number: 2018.68; date: 1 February 2018). The study was conducted in accordance with good clinical practice and the ethical standards outlined in the Declaration of Helsinki. Written informed consent was obtained from all participants prior to the study.

### 2.2. Subjects

Patients were recruited from an outpatient population from the Departments of Pulmonology in the Albert Schweitzer Hospital Dordrecht and in the Beatrix Hospital Gorinchem in the Netherlands between February 2018 till December 2018. Chronic HFNC therapy was indicated for patients diagnosed with COPD in accordance with the GOLD criteria, stage III–IV (FEV1 < 50%, FEV1/FVC ratio < 70%) [1]. Additional inclusion criteria included a high disease burden, defined as more than two exacerbations per year requiring treatment with corticosteroids or antibiotics [19] and one or more hospitalization in the past year, and being in a stable clinical condition. Patients were excluded if they had any lung or thoracic abnormalities other than COPD. Additional exclusion criteria included prior use of non-invasive ventilation (NIV), severe heart failure (New York Heart Association stage IV), obstructive sleep apnea and malignant comorbidities. Also, patients who had experienced acute respiratory failure or a COPD exacerbation within the 6 weeks prior to enrolment were excluded from the study [19]. 

### 2.3. Design and Measurements

**Figure 1:**
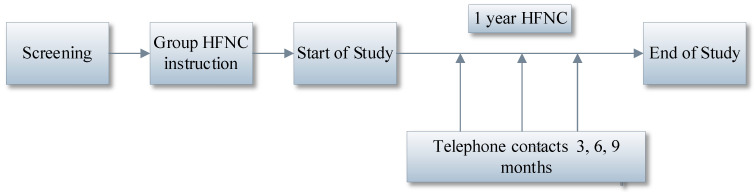
Flow Chart Design of the Study.

The study was designed as a prospective, proof-of-concept, interventional study. Eligible patients were given instructions on the usage of HFNC by a ventilation practitioner and research nurses during a group session. During this session an instructional video for the use of the HFNC was shown to patients and their care assistants. The compliance with HFNC usage was tested during each of the consecutive visits (see flow chart Figure 1).

Measurements, including demographics, comorbidities, body mass index (BMI), medication, lung function and blood gases, were taken at baseline before initiating HFNC treatment and again after 12 months of HFNC therapy. Health-Related Quality of Life (HRQoL) was assessed at each visit. Dyspnea was evaluated using the Medical Research Council‘s (MRC) 5-point dyspnea scale [20], and COPD control status was measured using the 7-point Clinical COPD Questionnaire (CCQ) [21]. The Hospital Anxiety and Depression Scale (HADS) (8 point per domain) was used to screen for symptoms of anxiety and depression [22]. 

#### 2.3.1. Lung Function

Lung function parameters were measured using the Masterlab-Compact^®^ system (Jaeger, Hochberg, Germany), in accordance with international guidelines [23]. Capillary blood gas measurements were obtained from the arterialized earlobe using the AVL Omni^®^ device (Roche Diagnostics GmbH, Graz, Austria). The partial pressure of carbon dioxide (pCO_2_) and bicarbonate concentration (HCO_3_^−^) were assessed using standard protocols.

#### 2.3.2. High-Flow Nasal Cannula Oxygen Treatment (HFNC)

The patients were admitted, according to a standardized protocol, by the same registered nurse trained on HFNC use. The HFNC device (Airvo-2™; Fisher & Paykel Healthcare, Auckland, New Zealand) consisted of a flow generator (up to 60 L/min), an air–oxygen blender that allows for adjustment of FiO_2_ from 21 to 100%, and an auto-fill MR 290 heated chamber. The gas mixture, kept at 34–37 °C, was delivered via a single-limb heated breathing tube to the patient via the Optiflow™ nasal cannula (Fisher & Paykel, Auckland, New Zealand) which was selected according to the size of the patients’ nostrils. The initial airflow was set at 25 L/min and adjusted according to patient tolerance. The HFNC was set to an absolute humidity of 44 mg H_2_O/L, temperature was set to 37 °C and FiO_2_ was adjusted to maintain an oxygen saturation as measured by a pulse oximetry (SpO_2_) of 88–92%. Patients were supported at home by nurses specialized in pulmonary diseases. In the event of pulmonary issues, patients could contact the hospital 24/7 by phone. During the follow-up interviews at 3, 6 and 9 monthas, detailed information was collected regarding any side effects of HFNC therapy. Patients were also able to visit the hospital at any time if needed. Compliance with HFNC usage was monitored through data obtained from the SD card readout of the Airvo-2™ device software.

### 2.4. Sample Size

An estimated total of 40 patients was needed, considering approximately 20% potential dropouts and patient recruitment feasibility. An estimated 32 study participants were needed to obtain an 80% power at a two-sided level of significance of 5% to detect a change in COPD exacerbation of 1 between the treatment periods, considering the results of previous studies [24]. We also assumed that the population standard deviation (SD) was 1.58, corresponding to a mean exacerbation count of 2.5 per year [24].

### 2.5. Statistical Analyses

Statistical analysis was performed using IBM SPSS version 23 software (IBM Corporation, Armonk, NY, USA). Repeated analysis of variance (ANOVA) measures were used to analyze the data. Significant effects identified in the ANOVA were further examined using Student’s *t*-tests. Differences within groups were assessed using two-tailed, paired t-tests, while differences between groups were evaluated with unpaired *t*-tests. Summarized statistics are presented as means ± standard deviation (SD). To examine the relationships between variables, Spearman‘s rank correlation tests were performed. Kaplan–Meier analysis was used to compare the time to first COPD exacerbation between periods. A *p*-value of <0.05 was considered statistically significant.

## 3. Results

**Figure 2:**
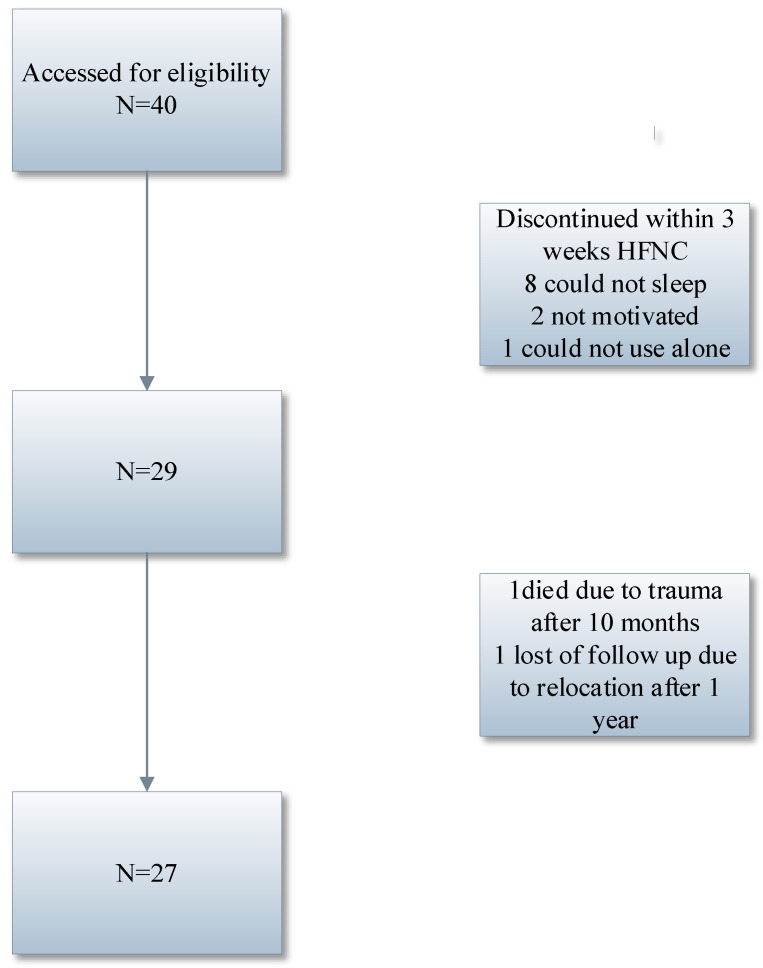
Flow chart of patient enrollment.

Forty patients were included in the study, 27 patients were evaluated after 1 year (Table 1). Eight patients did not endure treatment for the entire 3 weeks because of not being able to sleep with the HFNC. There were no exacerbations or hospitalizations during these periods. Two patients discontinued the study within 2 weeks because they were not motivated to continue with the treatment. There were no exacerbations or hospitalizations during these periods. One patient discontinued the study after 3 weeks since she could not cope with the HFNC. She could not start the HFNC by herself. No exacerbations or hospitalization during this period. One patient died after 10 months of treatment due to a trauma. She did not have exacerbations or hospitalization during this period. One patient succeeded in continuing the treatment for 1 year but there was no final visit because of their relocation. There were no exacerbations or hospitalization during this year of HFNC treatment (Figure 2). Seventeen patients were normocapnic with a capillary pCO_2_ < 6.0 kPa.

**Figure 3:**
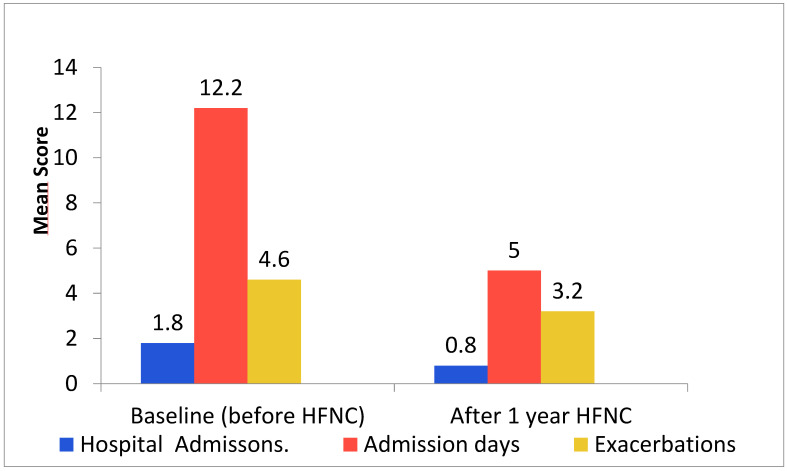
Mean hospital admissions, in-hospital days, and COPD exacerbation rate at baseline and after 1 year of HFNC treatment. HFNC; high-flow nasal cannula oxygen treatment, admission days; in-hospital days, exacerbations; COPD exacerbation rate.

Effects of HFNC on COPD exacerbation frequency, hospital admissions, in-hospital days, capillary pCO_2_ and Health Related Quality of life (HRQoL) measures:

The COPD exacerbation frequency rate decreased by 1.4 per year (mean difference ± SD: 1.4 ± 2.09; *p* = 0.002), hospital admissions reduced by 0.96 admissions (0.96 ± 1.37; *p* = 0.001). In hospital days were reduced by 7.2 days (7.2 ± 9.26; *p* = 0.001) (Table 2) (Figure 3). Capillary pCO_2_ was reduced by 0.02 kPa (0.02 ± 0.52: *p* = 0.85). The CCQ score declined by 0.06 (0.06 ± 0.96; *p* = 0.76). The MRC dyspnea score declined by 0.04 (0.04 ± 0.80; *p* = 0.81). The HADS anxiety score declined by 0.63 (0.63 ± 3.12; *p* = 0.31). The HADS depression score declined by 0.32 (0.32 ± 3.48; *p* = 0.64) (Table 2). 

**Figure 4:**
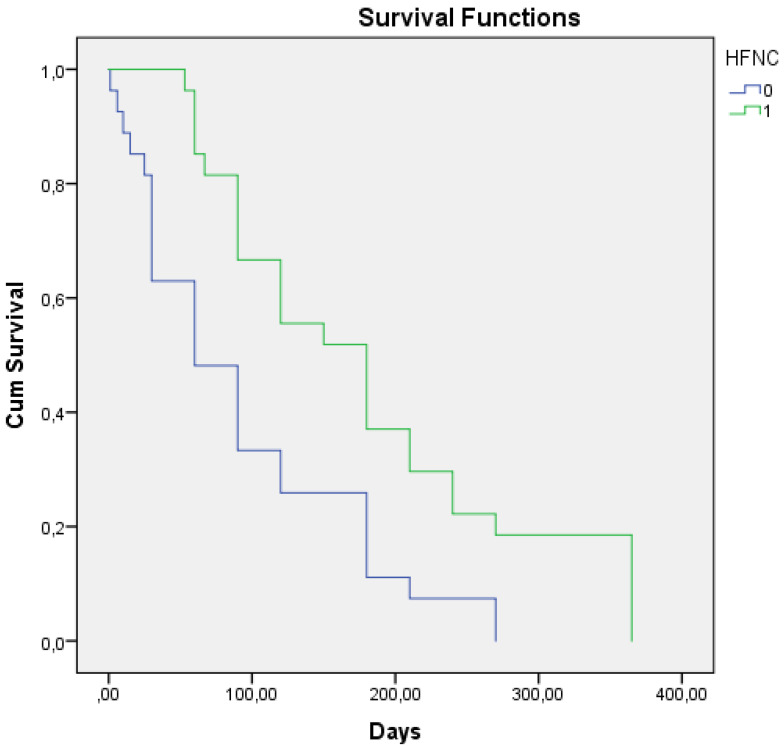
Kaplan–Meier curves for time to first COPD exacerbation one year before the start of high-flow nasal cannula (HFNC) oxygen treatment (blue line), and one year after start of HFNC oxygen treatment (green line). Mean time to first COPD exacerbation before HFNC treatment was 93.2 ± 15.4 days and after HFNC oxygen treatment it was 180.9 ± 20.8 days. The log-rank test showed a significance of *p* = 0.002.

Effects of HFNC oxygen treatment on time to first COPD exacerbation:

The mean time to first COPD exacerbation before HFNC treatment was 93.2 ± 15.4 days and after HFNC oxygen treatment it was 180.9 ± 20.8 days. The log-rank test showed a significance of *p* = 0.002 (Figure 4).

High-Flow Nasal Cannula Oxygen Treatment

HFNC settings were recorded properly at the start of treatment. After 12 months, the mean HFNC use time per night was 7.2 ± 1.5 h, and the mean flow rate used was 28.2 ± 1.9 L/min with a mean FiO_2_ of 28% and a mean temperature of 37 °C. The compliance with HFNC treatment did not differ between normocapnic and hypercapnic patients (*p* = 0.50) (Table 3).

Correlations between time of HFNC use and changes in hospital admission and symptoms scores.

There was no significant correlation between time of HFNC use and the decrease in the COPD exacerbation rate (correlation coefficient 0.13, *p* = 0.52) or the reduction in hospital admissions (correlation coefficient 0.44, *p* = 0.06) or in-hospital days (correlation coefficient 0.53, *p* = 0.06). Similarly, there were no significant correlations between the time of HFNC use and changes in symptom scores: CCQ score (correlation coefficient −0.35, *p* = 0.08), MRC dyspnea score (correlation coefficient 0.13, *p* = 0.50), HADS depression score (correlation coefficient 0.35, *p* = 0.07) or HADS anxiety score (correlation coefficient −0.02, *p* = 0.91). Nor was there a significant correlation between time of HFNC use and changes in capillary pCO_2_ (correlation coefficient 0.04, *p* = 0.83).

Differences in response between normocapnic and hypercapnic group

There were no statistically significant differences between the normocapnic and hypercapnic groups before the initiation of HFNC treatment regarding FEV1, FVC, MRC, CCQ, HADS anxiety and depression scores, age, BMI, COPD exacerbations, hospital admissions or in-hospital days (*p* > 0.05) (Table 1).

**Figure 5:**
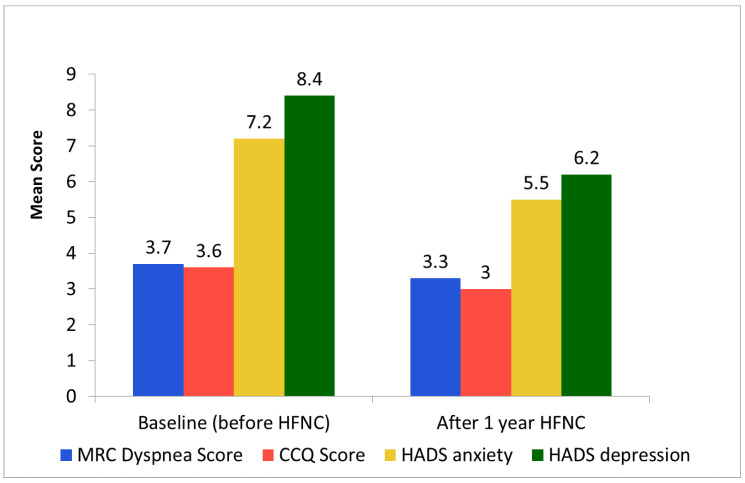
Mean MRC Dyspnea score, CCQ score and HADS score at baseline and after 1 year of HFNC treatment in the hypercapnic (pCO_2_ ≥ 6 kPa) group. HFNC; high-flow nasal cannula oxygen treatment, MRC; Medical Research Council, CCQ; Clinical COPD Questionnaire, HADS; Hospital Anxiety Depression Scale.

No statistical differences were observed between the normocapnic and hypercapnic groups in terms of changes in the COPD exacerbation rate (decreases of 1.82 ± 2.16 and 0.70 ± 1.88, respectively, *p* = 0.18), hospital admissions (decreases of 0.94 ± 1.34 and 0.90 ± 1.45, respectively, *p* = 0.94) or in-hospital days (decreases of 7.53 ± 7.79 and 5.60 ± 11.13, respectively, *p* = 0.64). Nor were statistical differences observed between the normocapnic group compared with the hypercapnic group in the change in capillary pCO_2_ (0.07 ± 0.47 and −0.06 ± 0.61 decrease, respectively, *p* = 0.53). However, a tendency for significant differences was observed between the groups in change in HRQoL symptoms scores with decrease in symptoms scores only in the hypercapnic group. MRC dyspnea score (−0.18 ± 0.53 and 0.40 ± 1.07 decrease, respectively, *p* = 0.07), CCQ score (−0.24 ± 0.55 and 0.49 ± 1.32 decrease, respectively, *p* = 0.05), HADS anxiety score (0.00 ± 3.46 and 1.70 ± 2.21 decrease, respectively, *p* = 0.17) and HADS depression score (−0.76 ± 1.86 and 2.20 ± 4.75 decrease, respectively, *p* = 0.03) (Figure 5).

Correlations between changes in COPD exacerbation rate, hospital admission and symptoms scores and changes in capillary pCO_2_

No significant correlation was observed between the change in capillary pCO_2_ (before and after 1 year of treatment) and the decrease in the COPD exacerbation rate (correlation coefficient 0.14, *p* = 0.48). Additionally, no significant correlation was found between the change in capillary pCO_2_ and the reduction in hospital admissions (correlation coefficient 0.32, *p* = 0.09) or in-hospital days (correlation coefficient 0.30, *p* = 0.12). Similarly, no significant correlations were observed between the change in capillary pCO_2_ and changes in symptom scores: CCQ score (correlation coefficient 0.19, *p* = 0.32), MRC dyspnea score (correlation coefficient 0.26, *p* = 0.19), HADS anxiety score (correlation coefficient 0.24, *p* = 0.22) or HADS depression score (correlation coefficient 0.35, *p* = 0.07).

## 4. Discussion

The present study demonstrated that home HFNC treatment reduced the number of COPD exacerbations, hospital admissions and length of hospital stays in severe COPD patients (GOLD III and IV) with frequent exacerbations and hospitalizations, regardless of hypercapnia. Furthermore, home HFNC improved HRQoL measures during the 1-year follow-up period, but only in patients with hypercapnia. This suggests that home HFNC therapy reduced the frequency of COPD exacerbations and hospital admissions in patients with severe COPD, irrespective of hypercapnia, highlighting the importance of considering factors beyond pCO₂ when deciding to give treatment to COPD patients with a home HFNC.

Patients with severe COPD (GOLD stages III and IV) often bear a substantial disease burden, with symptoms such as severe dyspnea, anxiety and a marked reduction in quality of life [25]. These patients frequently require hospitalization during exacerbations of their condition [26]. To our knowledge, this is the first study to investigate the effects of HFNC in patients with severe COPD, irrespective of having hypercapnia. The findings of this study align with the results reported by Raveling et al. [27] and Theunisse et al. [28] that non-invasive respiratory support like HFNC and NIV may have positive effects that go beyond their ability to reduce partial pressure of carbon dioxide (pCO_2_) in the blood. This suggests that the benefits of NIV or HFNC treatment should be examined more broadly, especially considering factors other than hypercapnia [27,28]. Furthermore, our findings are consistent with recent studies showing that long-term HFNC therapy effectively reduces COPD exacerbations and COPD-related hospital readmissions in patients with frequent exacerbations [18,24]. Similarly, our findings reproduced the results reported by Nagata et al. that HFNC prolonged the time to COPD exacerbation [18]. Our study emphasizes the results of the studies by Storgaard et al. [24] and Nagata et al. [29] that HFNC can improve symptoms and quality of life measures [24,29]. Similar to other studies, our study found no correlation between the decline in pCO_2_ and reductions in hospital admissions or symptom scores [24]. However, this study highlights that HFNC therapy is feasible for home use in patients with severe COPD, leading to improvements in symptoms and quality of life [17,24,29].

This study had several limitations. First, the lack of a placebo-controlled design is a presumed drawback. Although the usage of a placebo group with a sham device was contemplated, concerns about the validity of blinding arose, as both patients and investigators could potentially identify the sham intervention. Additionally, the use of a sham device was deemed impractical due to the challenges involved in blinding patients to the flow, heat and humidity associated with the treatment. Second, not every patient experienced an improvement with the treatment period. Eleven out of forty patients were unable to tolerate the HFNC therapy after 3 weeks. This high drop out in the first three weeks of treatment might be linked to a potential bias introduced by providing instructions on the use of HFNC during a group session. This group instruction session might not meet the expectations from patients and their care assistants. Nevertheless, this failure rate is consistent with those reported in earlier studies [24,30]. Despite this, the findings of this study confirm that long-term home HFNC management is feasible for patients with severe COPD on an outpatient basis [17,24,30,31]. Third, the mean flow (28.4 L/min) was relatively low in the present study. However, based on earlier studies, HFNC treatment should preferably be administered during sleep, with a flow rate of no less than 20 L/min [17,24]. Fourth, the duration of HFNC usage (7.2 h) could influence the outcome variables of the study. For a reduction in the COPD exacerbation rate, an average HFNC use of 6–7 h/day is recommended [24]. This suggests that the length of HFNC usage plays a crucial role in reducing COPD exacerbations. Furthermore, in a recent study, Sorensen et al. demonstrated that domiciliary HFNC use for more than 6 h is likely to be highly cost-effective for patients with severe COPD and persistent respiratory failure when compared to standard care [30]. Finally, using capillary blood sampling as a substitute for arterial blood sampling in a clinical setting could introduce bias. However, capillary blood sampling offers several advantages, including lower complication rates, reduced invasiveness, greater patient comfort and increased physician independence. In this study, capillary pCO_2_ was not significantly reduced by HFNC, but given that capillary pCO_2_ and HCO_3_^−^ levels have been shown to be accurate compared to arterial sampling methods [32], capillary blood sampling remains a valid approach in our study. Despite the fact that these limitations may have introduced some bias, the significant reductions in hospital admissions and COPD exacerbation rates across the entire group suggest that these factors did not substantially affect the overall findings. Therefore, we are confident that the results remain reliable despite these limitations. 

The main finding of this study is that in stable COPD patients, home HFNC reduces the numbers of COPD exacerbations, hospital admissions and in-hospital days. Furthermore, home HFNC improves quality of life measures only in the hypercapnic group of patients. Reducing symptoms and preventing exacerbations are considered key factors in slowing the progression of COPD [19]. One of the possible mechanisms behind these improvements is flushing of the anatomical dead space [33]. By using inhaled inert gas, HFNC treatment demonstrated a rapid clearance of the upper airway in a dose-dependent manner, with higher flow rates leading to greater clearance. Clearing CO_2_ from dead space can improve ventilator efficiency in test subjects [33,34], and therefore a reduction in the difficulty of breathing [35]. Another physiologic mechanism behind the improvements from HFNC treatment could be by generating a positive end-expiratory pressure (PEEP). The magnitude of PEEP measured is equal to the flow rate with an increase in PEEP of 0.7 cm H_2_O with every flow rate increase of 10 L/min [36]. As flow rates increase to 60 L/min, the PEEP can reach approximately 5 cm H_2_O when measured by esophageal manometry [37]. Another explanation for the positive effects of HFNC could be the improvement in mucociliary clearance. HFNC requires humidification and warming of the air to prevent drying of the mucous membranes. Humidification efficiency is notably enhanced at flow rates above 20 L/min [38]. To improve patient tolerability and ensure optimal humidification, HFNC systems are typically set to around 37 °C, which is close to the patient‘s core temperature. This warm, humidified air–gas mixture helps prevent dehydration of the mucus, thereby improving mucociliary clearance [39]. By improving mucociliary clearance, HFNC can reduce the risk of infections, which are thought to be an important origin of COPD exacerbations. All of the mechanisms could result in lesser workload [40] and consequently contribute to a better airflow redistribution in the lungs, thereby improving ventilation–perfusion matching [34]. The recovery of the ventilation–perfusion matching was positively correlated with both the anxiety domain of the Severe Respiratory Insufficiency Questionnaire and the 6-minute walking test [41]. A combination of these mechanisms would lead to a decrease in patients’ sensation of dyspnea. This improvement in dyspnea could, in turn, enhance their physical ability and, as previously indicated, reduce exacerbations [19]. Furthermore, a recent study by Raveling et al. found that the beneficial effect on HRQoL is achieved only partially through a reduction in pCO_2_, strengthening the evidence for a multifactorial origin of the beneficial effect of home NIV treatment like HFNC [42].

## 5. Conclusions and Implications

This proof-of-concept multicenter study using home HFNC in severe COPD patients with frequent exacerbations, regardless of hypercapnia status, demonstrated reductions in COPD exacerbation rates, hospital admissions and in-hospital days. Furthermore, home HFNC treatment improved symptoms and quality of life measures, but these benefits were most pronounced in COPD patients with hypercapnia. Although based on a proof-of-concept study, these findings may imply that patients with severe COPD (GOLD stages III and IV) with frequent exacerbations, regardless of hypercapnia, may be suitable candidates for home HFNC therapy. In further investigations, a randomized controlled trial focusing on the use of home HFNC therapy for severe COPD patients with frequent exacerbations and hospitalizations, irrespective of hypercapnia, could provide valuable insights into whether this therapy can improve clinical outcomes. The study would help determine if home HFNC therapy is an effective, safe and cost-efficient way to manage these patients outside the hospital setting, potentially reducing exacerbations and hospital admissions and improving quality of life.

## Figures and Tables

**Table 1:** Clinical and demographic characteristics of patients at baseline.

N = 27	Mean (SD)	Normocapnic N = 17 Capillary pCO_2_ < 6.0 kPa	Hypercapnic N = 10 Capillary pCO_2_ ≥ 6.0 kPa	*p*-Values
Age, years	68.3 (7.8)	69.8 (7.6)	65.7 (7.7)	0.50
Male, %	48.1	58.8	30	0.24
Body-mass index, kg/m^2^	25.3 (4.0)	26.6 (4.2)	23.0 (4.0)	0.45
Normocapnic, % (capillary pCO_2_ < 6.0 kPa)	63	100	0	
FEV_1_, liters	0.99 (0.33)	1.1 (0.4)	0.80 (0.2)	0.06
FEV_1_ predicted, %	37.1 (8.6)	39.6 (8.5)	32.8(7.3)	0.22
FVC, liters	2.68 (0.84)	2.86 (0.9)	2.38 (0.7)	0.84
FVC predicted, %	83.8 (22.2)	87.0 (22.1)	78.3 (22.5)	0.93
FEV1/VC	38.5 (10.7)	39.8 (11.0)	36.1 (10.4)	0.97
pCO_2_ capillary, kPa	5.8 (1.0)	5.4 (0.5)	6.6 (1.2)	0.11
HCO_3_^−^ capillary, mmol/L	27.8 (3.0)	26.6 (2.3)	30.1 (2.8)	0.25
COPD exacerbation	4.6 (2.6)	4.8 (2.4)	4.3 (2.9)	0.35
Hospital admissions (1 year before HFNC)	1.78 (0.9)	1.7 (0.8)	1.9 (1.0)	0.77
In hospital days (1 year before HFNC)	12.2 (6.9)	11.1 (6.0)	14.2 (8.2)	0.17

**Note:** Data presented are mean (SD) unless otherwise stated. **Abbreviations:** FEV_1_, forced expiratory volume in 1 second; FVC, forced vital capacity; HCO_3_^−^, capillary bicarbonate; pCO_2_, carbon dioxide pressure.

**Table 2:** Hospital admissions, symptoms scores and pCO_2_ before and after 1 year of HFNC treatment.

N = 27	1 Year Before, Mean (SD)	1 Year After, Mean (SD)	Mean Difference (SD)	*p*-Value
COPD exacerbations	4.62 (2.63)	3.22 (1.74)	1.41 (2.09)	0.002
Hospital admission	1.78 (1.93)	0.81 (1.18)	0.96 (1.37)	0.001
In hospital days	12.19 (6.91)	4.96 (8.16)	7.22 (9.26)	0.001
MRC scores	3.00 (1.07)	2.96 (1.09)	0.04 (0.80)	0.81
CCQ scores	3.19 (1.04)	3.13 (1.24)	0.06 (0.96)	0.76
HADS anxiety	6.85 (4.95)	6.22 (4.98)	0.63 (3.12)	0.31
HADS depression	7.37 (5.02)	7.05 (5.20)	0.32 (3.48)	0.64
Capillary pCO_2_ kPa	5.87 (0.99)	5.85 (1.09)	0.02 (0.52)	0.85

**Note:** Data presented are mean (SD) unless otherwise stated. **Abbreviations:** CCQ, Clinical COPD Questionnaire scores; HADS, Hospital Anxiety and Depression Scale; MRC, Medical Research Council dyspnea scores; pCO_2,_ carbon dioxide pressure.

**Table 3:** HFNC usage and setting.

N = 27		Mean (SD)
Predominant Use Night	(hours)	7.2 (1.5)
Flow rate	L/min	28.2 (1.9)
FiO_2_	%	28
Temperature	°C	37

**Note:** Data presented are mean (SD) unless otherwise stated. **Abbreviations:** HFNC, high-flow nasal cannula oxygen therapy.

## Data Availability

The dataset consisting of de-identified participants‘ data is available from the corresponding author upon reasonable request.
